# The Impact of Physical Props and Physics-Associated Visual Feedback on VR Archery Performance

**DOI:** 10.3390/s25226991

**Published:** 2025-11-15

**Authors:** Zhenyu Liu, Haojun Xu, Mengyang Tu, Feng Tian

**Affiliations:** Shanghai Film Academy, Shanghai University, Shanghai 200072, China; cloudmoon@shu.edu.cn (Z.L.); sacross_k@shu.edu.cn (H.X.); 1521427606@shu.edu.cn (M.T.)

**Keywords:** virtual reality, physical prop interfaces, visual feedback, motor performance, user experience

## Abstract

**Highlights:**

**What are the main findings?**

- Physical props significantly enhance presence and enjoyment but increase hand tremor, impairing task performance in high-skill VR archery.
- Physics-associated visual feedback moderates the negative impact of hand tremor on performance and synergistically enhances flow experience when combined with physical props.

**What are the implications of the main findings?**

- VR designers should implement multimodal integration where physical props are paired with congruent visual feedback to optimize both engagement and performance.
- The identified moderated pathway provides a framework for balancing experiential benefits against performance demands in high-skill VR applications.

**Abstract:**

Most existing virtual reality exergames rely on generic VR devices, which can limit the physical exertion in VR-based exercises. In contrast, physical props can enhance exercise intensity, yet their impact on users’ performance and experience remains understudied, particularly in skill-based tasks. Meanwhile, physical props offer richer tactile and kinesthetic feedback, which, combined with the visual effects of head-mounted displays, presents a potential solution for improving user experience in VR. To explore this, this study developed a sensor-driven experimental framework for investigating high-skill VR tasks. By integrating vision sensors with standard VR devices, we constructed a VR archery system that enables objective quantification of motor performance. Leveraging the sensor-driven framework, we investigate the effects of physical props and physics-associated visual feedback on players’ performance and experience in VR tasks through an experiment involving 33 participants. By objectively quantifying performance, we reveal a dual-pathway mechanism: physical props significantly increased hand tremor, which in turn impaired aiming accuracy, but this negative effect was effectively moderated by time and physics-associated visual feedback that enabled real-time sensorimotor compensation. While complex physical props reduced task performance, they substantially enhanced enjoyment and presence, particularly demonstrating a synergistic effect on users’ flow experience when combined with physics-associated visual feedback. These findings elucidate the complex interplay between physical prop interfaces and visual feedback in high-skill VR tasks, providing valuable insights for designing VR experiences which balance performance requirements and engagement enhancement.

## 1. Introduction

### 1.1. Background

Virtual reality (VR) interaction often relies on generic devices like standard controllers, which significantly limit the intensity, realism, and fidelity of physical activities within virtual environments, particularly in high-skill domains [1]. While these devices offer versatility, they fail to replicate the nuanced movements and rich tactile sensations required for real-world skilled tasks, creating a gap in functional fidelity [2].

To address this limitation, researchers have explored specialized haptic interfaces and physical props [3]. Evidence suggests that realistic interfaces, such as physical props used in sports simulations, can provide superior kinesthetic feedback, potentially enhancing motor learning and performance in VR [4]. However, the impact of physical props on high-skill tasks presents a complex trade-off: while they may enhance sensory engagement, they could simultaneously increase motor control demands—a tension that remains inadequately understood. While there is already considerable research on physical props in virtual reality [5], few studies have explored the multimodal combination of physical props and visual feedback associated with them.

The multimodal nature of VR further complicates this relationship. Users inherently integrate sensory information from multiple channels [6,7], making the interplay between haptic and visual cues particularly important. Physics-associated visual feedback—where visual cues are dynamically tied to the physical state of a prop—represents a promising approach to enhance interaction realism. Nevertheless, existing evidence on multimodal integration remains conflicted. Some studies report synergistic benefits for both experience and performance [8,9,10,11], while others indicate negligible effects or even cognitive overload [12,13,14,15]. This inconsistency may stem from a predominant focus on main effects, overlooking the underlying psychophysiological mechanisms—such as sensorimotor integration and compensatory motor control—in high-skill task contexts.

Archery serves as an ideal paradigm for investigating VR interaction and immersion. It inherently combines vibrotactile, force, and kinesthetic feedback, demanding high levels of precision, force control, and adaptation [16,17]. Its established use in both VR training [18,19,20,21] and entertainment [22] provides a robust foundation for this study. Specialized hardware interfaces are pivotal for creating realistic VR archery experiences. Previous research has explored diverse technological pathways. For instance, some systems use a real recurve bow integrated with multiple sensors (e.g., reed sensors, laser, Kinect) to detect drawing and releasing, but at the cost of increased system complexity and setup [23]. Others employ high-precision custom devices with strain gauges, focusing on physical accuracy over immersive experience [24]. Conversely, approaches using data gloves or purely virtual interactions avoid physical props altogether, thereby lacking realistic tactile resistance feedback [25]. Crucially, there is a lack of validation regarding how these physical prop interfaces and their associated sensor modalities concretely impact user performance and experience.

To systematically investigate the impact of physical prop interfaces and visual feedback in high-skill VR tasks, this study establishes a sensor-driven experimental framework for VR archery—a task requiring precise motor control, force management, and stability. Within this framework, we address three research objectives:

- Develop and validate a virtual reality archery framework with integrated sensors that can objectively quantify motion performance through data acquisition.
- Investigate the underlying mechanisms through which physical props affect both performance and user experience in high-skill VR tasks.
- Explore how does physics-associated visual feedback interact with the physical prop interfaces, and can it mitigate potential performance decrements while enhancing the subjective experience.

To answer these questions, we conducted a 2×2 factorial experiment (Input interfaces: Physical Prop vs. Standard Controller × Visual Feedback: Physics-associated vs. Static) with 33 participants. The framework enabled objective quantification of motor performance through sensor-derived measures (hit deviation, hand tremor, completion time), complemented by multi-faceted subjective experience assessments (presence, flow, competence). Crucially, we employed linear mixed-effects modeling to unravel the pathway from the physical prop interfaces to hand tremor to performance accuracy, and to test the moderating role of visual feedback in this chain. This work makes three key contributions.

- We establish a sensor-driven experimental framework that objectively quantify motor performance in VR archery tasks, offering a potential pathway for methodological refinement in XR interaction research.
- Through objective quantitative metrics, this study provides a nuanced, mechanism-based explanation for the performance-experience trade-off associated with physical props in high-skill VR tasks.
- This study elucidates how physics-associated visual feedback moderates the impact of motor instability on performance and experience, offering principled insights for designing effective and engaging multimodal VR interactions.

### 1.2. Related Work

#### 1.2.1. Application Research of Physical Props in VR

Physical prop interfaces can be implemented through various methods. Beyond simulating vibrations via handheld controllers, recent research has proposed diverse approaches to enhance the realism and interactivity of virtual experiences. As indicated in the background, the use of physical props is a prominent approach to enhancing kinesthetic feedback in VR. In this section, we review relevant work in this area in detail.

Cheng explored the use of passive props to augment VR experiences [5], proposing that even non-actuated haptic props can heighten immersion. Their results indicated that passive props, despite lacking active feedback, improve users’ perception of virtual environments through physical presence, yielding more enjoyable and authentic experiences compared to scenarios without kinesthetic feedback.

White further investigated the effects of weighted physical prop interfaces on user experience and task performance [26]. Their experiments demonstrated that weighted props outperform conventional controllers in enhancing immersion, flow, and gameplay satisfaction, suggesting that physical weight alone can significantly elevate virtual experiences for specific tasks.

Strandholt introduced a redirected mapping technique to address insufficient haptic feedback [27]. By warping the relationship between physical and virtual tools, researchers enabled physical props to simulate impact and resistance when interacting with virtual objects. Their study revealed that redirected mapping significantly enhances interaction realism without compromising precision, underscoring the importance of mapping accuracy between virtual tools and physical props for user experience.

Auda conducted user experiments to examine the alignment between virtual objects and physical props of varying sizes [28]. Their findings suggest that precise size matching between virtual and physical objects is unnecessary for certain tasks. Most recently, Mori demonstrated that the physical properties of props play a critical role in improving immersion and applicability [29]. Collectively, existing studies confirm that haptic feedback via physical props effectively enhances immersion in VR. Different feedback modalities—such as length, weight, and active versus passive feedback—may yield varied outcomes across virtual tasks. While implementation specifics differ (e.g., precision in virtual-physical object alignment or feedback types), these works collectively validate the pivotal role of physical props in VR interaction and offer multiple pathways for advancing related research. However, none of the above studies explore the multimodal combination of physical props and visual feedback associated with them.

#### 1.2.2. The Impact of Simulated Haptic and Visual Feedback in VR

To address the research gap concerning the interaction between haptic interfaces and visual feedback in high-skill tasks, it is essential to review existing evidence on multimodal integration.

Early foundational work [30] found that augmented visual feedback accelerates learning, particularly in coordination tasks within virtual environments. However, some argued that frequent augmented visual feedback in procedural skill learning does not improve outcomes and may even hinder learning [14]. This could stem from the cognitive overload caused by excessive sensory information [15], underscoring the importance of feedback intensity modulation in VR, especially across varying task demands.

The congruency between sensory modalities has emerged as a critical factor. Williams examined the effects of incongruent visual feedback (relative to real-world operations) in VR racing simulations [31]. Their results revealed that inconsistent visual feedback primarily alters user perception rather than directly impairing task performance. Gibbs conducted experiments where participants interacted with a virtual ball bouncing on a virtual rod [32]. They found that simultaneous visual and haptic feedback amplified the sense of presence, demonstrating the synergistic benefits of multimodal fusion.

Conversely, Gao developed a virtual pottery [33], demonstrating that haptic feedback combined with high-fidelity visual feedback markedly improves operational accuracy, more than just presence, compared to bare-hand interactions. Collectively, these studies emphasize the critical role of congruency between visual and haptic feedback in optimizing VR experiences and performance.

Despite the widespread acknowledgment of haptic feedback’s utility in virtual environments [3], discrepancies persist across studies regarding feedback intensity, modality types, and the efficacy of multimodal integration. The effectiveness of haptic feedback appears contingent not only on its presence but also on task complexity, feedback intensity, and perceptual congruency.

The efficacy of feedback is further moderated by task complexity. Supporting this view, Van demonstrated that simulated vibrotactile feedback significantly enhanced performance in complex tasks under latency but had negligible effects on simple tasks [34]. Likewise, the intensity of the feedback itself is a key variable; Tanacar highlighted that higher-intensity haptic rendering could lead to lower error rates [35], indicating that the potency of the signal influences performance outcomes.

Finally, moving beyond performance and subjective experience, some research has begun to explore psychophysiological mechanisms. Radhakrishnan investigated the relationship between haptic feedback [36], fine motor skill training, and physiological arousal, suggesting that haptic feedback may influence arousal levels even in the absence of significant changes in self-reported measures like presence or task load.

In summary, despite the acknowledged utility of multimodal feedback, notable discrepancies persist across studies. The literature suggests that its effectiveness is contingent on a nuanced interplay of factors, including task complexity, feedback intensity, and, crucially, perceptual congruency—a finding that directly informs the design of our experiment within the high-skill context of archery.

## 2. Material and Methods

### 2.1. Design of the Physical Prop Interfaces

To achieve our experimental goals, we designed a VR platform simulation application for archery. Through this application, we can provide users with a realistic simulation archery experience. The application was developed using the Unity3D engine and can run on the Meta Oculus 3 platform. The application offers two main operating modes. The first one uses a standard controller, which is consistent with common VR archery games. Users can use the standard controller interface to input operations. One controller is used to operate the position of the bow, and the other controller’s grab key is used to perform the action of pulling and releasing the bowstring.

**Figure 1:**
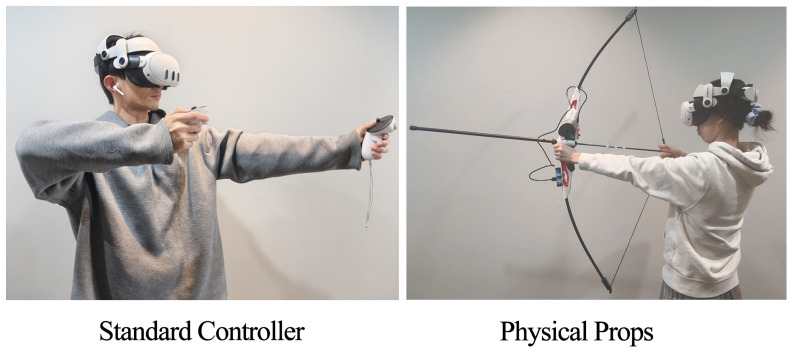
Two types of interaction devices.

The second approach is to use a physical props interface based on simulated bows and arrows, as shown in Figure 1. Users can directly operate a simulated bow, and the location, shape, and function of this simulated bow will be synchronized and mapped in real time to the virtual bow in the virtual space.

#### 2.1.1. The Hardware Architecture

**Figure 2:**
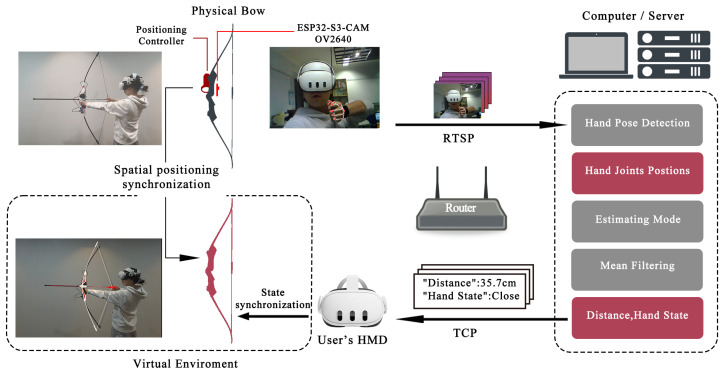
The structure of physical prop interfaces for virtual archery.

The system’s hardware architecture extends beyond the VR head-mounted display and standard controllers. Additional components critical to the physical prop mode include a simulated physical bow (660 g, 10 lbs), an ESP32S3Cam microcontroller board, an OV2640 camera module, and a battery component. Figure 2 illustrates the hardware configuration. To ensure spatial consistency between the real-world bow and its virtual counterpart, the standard controllers are securely affixed to the bow’s frame as positioning trackers, with their relative positions mapped in the virtual environment. Additionally, an OV2640 camera, integrated with an ESP32S3Cam microcontroller, is mounted at the center of the physical bow and oriented toward the user for real-time hand motion capture. The entire module is powered by an independent battery component.

The video stream is transmitted via the Real-Time Streaming Protocol (RTSP) to a host platform, where a task-specific neural network processes the data to identify hand posture and spatial positioning. Predicted data frames undergo temporal smoothing and anti-jitter filtering before being relayed back to the client through the Transmission Control Protocol (TCP), guiding corresponding actions of the virtual bow.

#### 2.1.2. Gesture Recognition

**Figure 3:**
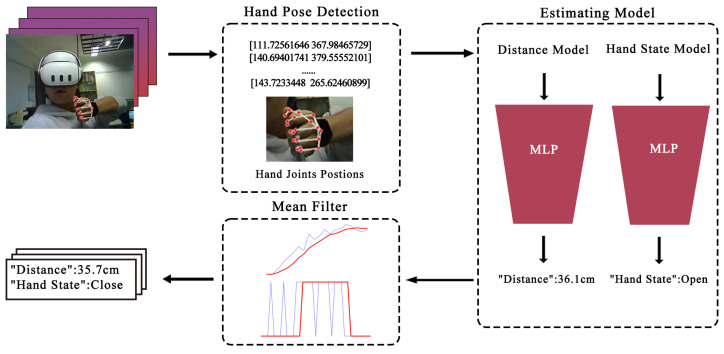
The structure of Gesture recognition. The raw data stream from the MLP models (shown as the blue line) contains inherent noise and jitter. To address this, we apply a mean filter, which produces the stabilized signal (shown as the red line).

To enable interaction by capturing users’ natural gestures, we developed a gesture recognition system as shown in Figure 3. This system was designed to accurately track hand positions during bow-drawing and release phases. The technical pipeline consisted of three main stages: camera calibration, synthetic dataset generation, and model training and deployment.

Camera Calibration: The OV2640 camera module was calibrated using a standard checkerboard pattern to obtain its intrinsic parameters, distortion coefficients, and resolution. These calibrated parameters were subsequently used both in the synthetic data generation process and to correct image distortion in the live video stream.

Synthetic Dataset Generation: To train the recognition model, we created a custom synthetic 3D hand joint pose dataset. We first collected several common archery drawing hand postures (e.g., Mediterranean release, Mongolian release) and release states, recording their 3D joint spatial coordinates. These were categorized into two classes: “Closed” for drawing postures and “Open” for other states. A virtual camera, parameterized with the real camera’s calibrated intrinsics, distortion coefficients and resolution, was positioned at the origin in 3D space. We then randomly sampled hand poses, distances, and rotations, ensuring the hand remained in front of the virtual camera’s field of view. The 3D joint coordinates were projected onto the 2D image plane using the virtual camera’s parameters. The Euclidean distance between a predefined index finger joint and the camera was computed as the ground-truth depth. This process generated a rich synthetic dataset containing tuples of (3D joint coordinates, virtual transformation, 2D projected joint coordinates, true distance, gesture state).

Model Training and Performance: Two independent Multilayer Perceptron (MLP) networks were trained on this dataset: one for hand posture classification (“Open”/“Closed”) and another for distance regression. The input to both networks was the vector of 2D joint coordinates from a single frame. We employed an 80:20 random split for training and testing. The distance regression model achieved a Mean Absolute Error (MAE) of less than 2 cm on the test set, which corresponds to a relative error of approximately 4% given the typical draw distance range of 45–50 cm. The binary posture classification model achieved an accuracy of approximately 95% on the test set. The model’s prediction latency is approximately 30 ms.

System Deployment and Workflow: In the operational system, the OV2640 camera, mounted at the center of the physical bow and oriented toward the user, captures real-time hand images at 20–25 frames per second. The video stream is transmitted via RTSP to a host platform. Each frame is processed by the trained MLP networks to simultaneously predict the hand posture state and the longitudinal draw distance. The predicted data frames undergo temporal smoothing and anti-jitter filtering before being relayed back to the VR client via TCP, where they drive the corresponding actions of the virtual bow (e.g., drawing the string, calculating draw force). The end-to-end latency of this pipeline, from image capture to action in VR, is approximately 100 ms. The resulting system usability scores (reported in Section 3.2.5) confirm that this computer vision-based approach maintains a good level of usability for the interactive task.

### 2.2. Physics-Associated Visual Feedback

**Figure 4:**
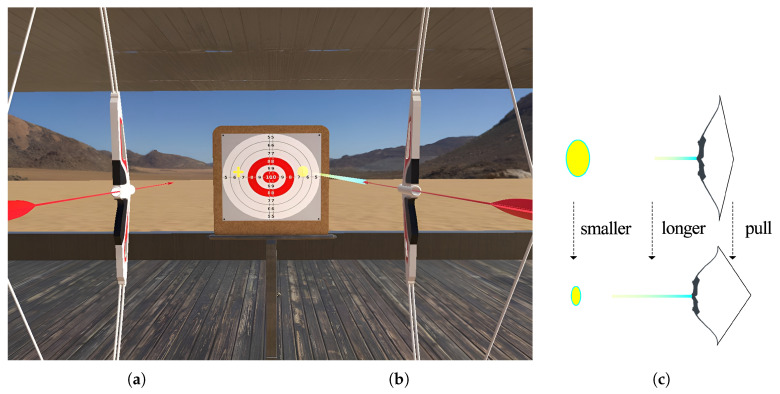
Two types of visual feedback. (**a**) The type of visual feedback with a small static crosshair. (**b**) The type of visual feedback with a dynamic circular reticle. (**c**) The trajectory line length and reticle radius are dynamically changing. The dashed lines illustrate the transition process of the trajectory extension and reticle scaling, with the blue-to-yellow gradient line representing the trajectory ray and the yellow ellipse indicating the reticle on the target.

The system implements a physics-associated visual feedback mechanism. In the absence of physics-associated visual feedback, a small static crosshair is rendered at the intersection of the arrow’s straight linear trajectory and the target plane (Figure 4a) to ensure objective aiming fairness.

When physics-associated visual feedback is enabled, during the aiming phase, the system generates a semitransparent trajectory line at the arrow tip, which dynamically extends along its straight path. This trajectory line is completely straight and unaffected by simulated gravity or wind. Simultaneously, a circular crosshair is displayed at the intersection of this trajectory line and the target plane (Figure 4b). The length of the trajectory line and the radius of the crosshair are dynamically adjusted according to the draw distance: as the draw distance increases, the trajectory line visually lengthens, while the radius of the crosshair decreases (Figure 4c). This real-time synchronization ensures that the visual feedback is consistent with the physical force feedback originating from the bow, hence the term physics-associated visual feedback. Specifically, the draw distance is mapped to the pull of the virtual bow, which in turn affects the simulated speed and trajectory of the arrow. The actual trajectory of the arrow is affected by gravity and wind, so the trajectory shown in the visual feedback is completely different from the actual trajectory of the arrow. Participants need to use their skills to predict the movement of the arrow, which is the main difficulty of this archery task.

When users engage in the aiming task, their visual focus centers on the reticle and target area rather than the virtual bow model. In static crosshair mode, users perceive only tactile feedback from the standard controller or physical prop. In dynamic feedback mode, while the reticle’s positional alignment matches the static version, users observe additional dynamic visual cues (e.g., trajectory line and reticle scaling) that are directly mapped to the physical prop’s real-time state. This multimodal integration establishes a closed-loop connection between visual and kinesthetic feedback, reinforcing sensory congruency and consistency.

### 2.3. Experiment

#### 2.3.1. Study Design

Based on our two distinct interaction devices and visual feedback configurations, the system enables combinations of varying interaction modalities. Under these conditions, we measured participants’ sense of presence, physical activity enjoyment (PAE), task load, self-efficacy, and other subjective metrics while simultaneously recording objective behavioral data in the virtual environment.

The experiment employed a 2×2 factorial design with two independent variables: haptic interface type (standard controller [SC] vs. physical prop [PP]) and physics-associated visual feedback (absent vs. present). The four experimental conditions were (1) SC without VF (SC − VF), (2) SC with VF (SC + VF), (3) PP without VF (PP − VF), and (4) PP with VF (PP + VF). A balanced Latin square design [37] was implemented to counterbalance the order of conditions across participants.

#### 2.3.2. Participants

A total of 33 adult participants were recruited from the university population. Informed consent was obtained from all subjects involved in the study. The participants had a mean age of 23.8 (±4.4) years, with a minimum age of 20. Only one participant reported extensive prior experience in archery, while two had never used VR devices.

#### 2.3.3. Procedures

We invited volunteers to participate in the experiment under four distinct conditions. Initially, we provided a detailed explanation of the simulated bow system’s interaction methods and task rules. Prior to participation, all volunteers underwent an informed consent process, which involved reading and signing an informed consent form.

The sequence in which volunteers experienced the different modes was determined using a Latin square design to control for order effects. Before commencing each mode, participants were required to familiarize themselves with the respective interaction method through practice sessions. These practice sessions allowed participants to conduct trial shots (a minimum of 10 practice shots were required, and participants could take more if they felt necessary) to ensure they were comfortable and proficient with the interaction mechanics before proceeding to the formal experimental trials. Participants are required to have sufficient rest between the practice and formal phases.

In the formal experimental phase, participants engaged in archery tasks using either two standard controller or a physical prop. Their primary objective was to achieve maximum accuracy by aiming at the target while accounting for wind direction (left or right) and the gravitational effects on the arrow. Upon hitting the target or the plane where the target is located, participants received a score ranging from 0 to 10 based on the proximity of the impact point to the center of the target. The wind direction changed after each shot, and participants could consult a user interface panel to obtain real-time information about the current wind speed, wind direction and score.

We introduced the wind factor primarily to enhance the task’s challenge and realism. To ensure experimental control, wind direction and speed were randomized during practice trials but followed a pre-defined sequence across the 10 formal shots for all participants. The sequence ensures a balanced distribution of wind direction and intensity in both the left and right directions and in terms of intensity level. This design ensured consistent wind conditions for each corresponding shot number. It remained constant and identical across all experimental groups.

Each mode consisted of 10 formal shooting trials per participant to ensure that performance metrics reflected a stable representation of their abilities within that mode without inducing excessive fatigue. The number of tests was determined based on our preliminary experiments, where we found that more than 15 consecutive shots were generally difficult for the average adult to complete. Although we allowed participants to rest at any time, we chose a number of shots that would not cause excessive fatigue for most adults to avoid the impact of frequent interruptions on their subjective experience. Following the completion of the experimental trials, participants completed an evaluation questionnaire designed to assess their experience with each interaction mode.

#### 2.3.4. Measures

To comprehensively evaluate players’ performance and experience, we collected both objective behavioral data and subjective questionnaire responses. The measurement variables are summarized in Table 1 and described in detail below.

##### Objective Measures

To provide quantitative evidence of motor performance, we employed a sensor data acquisition approach. We derived three key performance metrics from this sensor-driven framework that capture different aspects of task execution in VR archery. These metrics were chosen to provide a comprehensive assessment of users’ motor performance, stability, and efficiency during the aiming and shooting process.

1. Hit deviation, which reflects the precision with which users completed the task. For each shot, the Euclidean distance between the arrow’s impact coordinates on the target plane and the bullseye is recorded. This distance is then normalized using the radial separation from the bullseye (10-ring) to the 5-ring line as one unit.
2. Hand tremor, an objective factor potentially affecting precision, was operationalized as the mean positional deviation of the bow. This metric was derived from continuous sampling of the bow’s position at 60 Hz in the second before firing. For each frame, the Euclidean distance from the bow’s position to the average center across all frames was computed. The arithmetic mean of these distances (in centimeters) was then used as the quantification index, with higher values denoting poorer arm stability.
3. Task completion time, which indicates efficiency. For each shot, the system records the precise duration from the initiation of the aiming action to the moment the arrow is released.

##### Subjective Measures

We opted to select specific dimensions from multiple scales to measure subjective experience rather than directly using the Game Experience Questionnaire(GEQ) [38]. The GEQ is widely used in game experience research, it is designed to cover various types of gaming experiences. In contrast, VR environments are relatively unique, and archery tasks are more akin to physical sports rather than purely electronic games. Therefore, for different dimensions such as presence, we selected the following measurement tools.

1. Presence: We selected 4 items from the I-group Presence Questionnaire [39] to assess the immersive experience of the players.
2. Physical Activity Enjoyment: We selected 4 items from the Short Version of the Physical Activity Enjoyment Scale [40] to evaluate the enjoyment of the players, focusing on positive experiences during the activity.
3. Flow experience: We selected 4 items from a validated scale [41] to measure the flow experience of the players, covering key dimensions such as concentration and sense of control.
4. Competence: To assess players’ sense of competence—a psychological need reflecting their perceived effectiveness and mastery in the game environment—we selected 4 items from the Competence subscale of the Game Experience Questionnaire [38].
5. Task Load: We measured the mental and physical demands of the task using the 6 items of the NASA-TLX questionnaire [42], and the overall workload score was computed using the unweighted Raw TLX procedure.
6. System Usability: We evaluated the usability of the game system using the 10 items of the System Usability Scale [43].
7. Future Use Intention: We measured players’ intention to continue using the system in the future through a single-item scale, reflecting the system’s attractiveness and potential value.

## 3. Results

### 3.1. Task Performance

**Figure 5:**
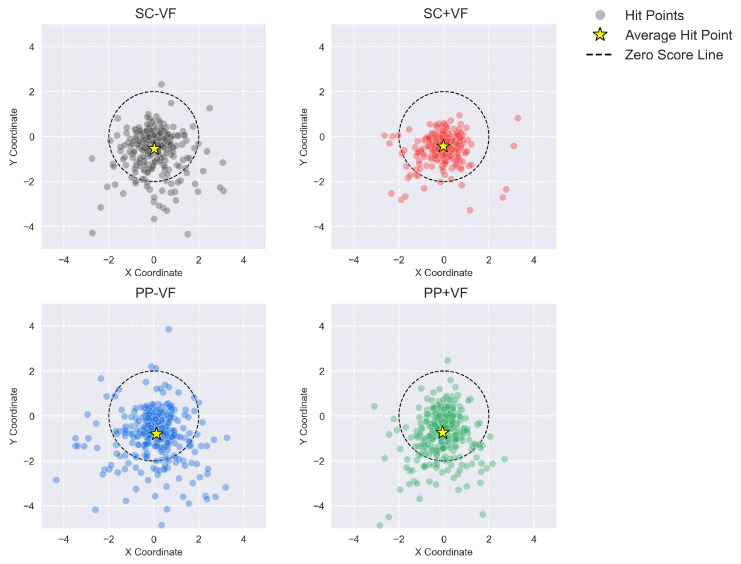
The distribution of all hit deviations across all conditions.

In this study, we conducted comprehensive statistical analyses of participants’ performance across four experimental conditions, encompassing objective metrics such as hit deviation, task completion time, and hand tremor. To ensure data representativeness and accuracy, all statistical evaluations were based on data from all individual shooting trials during the formal experiment. We collected 33 sets of objective behavioral data from 33 participants, including 10 shooting trials per participant under each condition. The mean hit deviation across each participant’s ten shots was utilized to represent their personal hit deviation and included in subsequent statistical analyzes. Similarly, the mean task completion time and mean hand tremor across the ten trials were calculated for each participant and used in the statistical analyses. Figure 5 illustrates the distribution of all raw hit deviations (i.e., every individual shooting trial across all participants and conditions). It can be observed that the border of the hit deviations is generally wider in the physical prop conditions (PP − VF and PP + VF) compared to the standard controller conditions (SC − VF and SC + VF). Moreover, the presence of visual feedback seems to reduce the border of the hit deviation in the standard controller condition (SC − VF vs. SC + VF), and the observed difference is weaker under the physical prop condition (PP − VF vs. PP + VF). However, the differences we observed were mainly reflected in a small number of cases with large deviation values, which does not represent the overall situation.

#### 3.1.1. Hit Deviation Analysis

**Figure 6:**
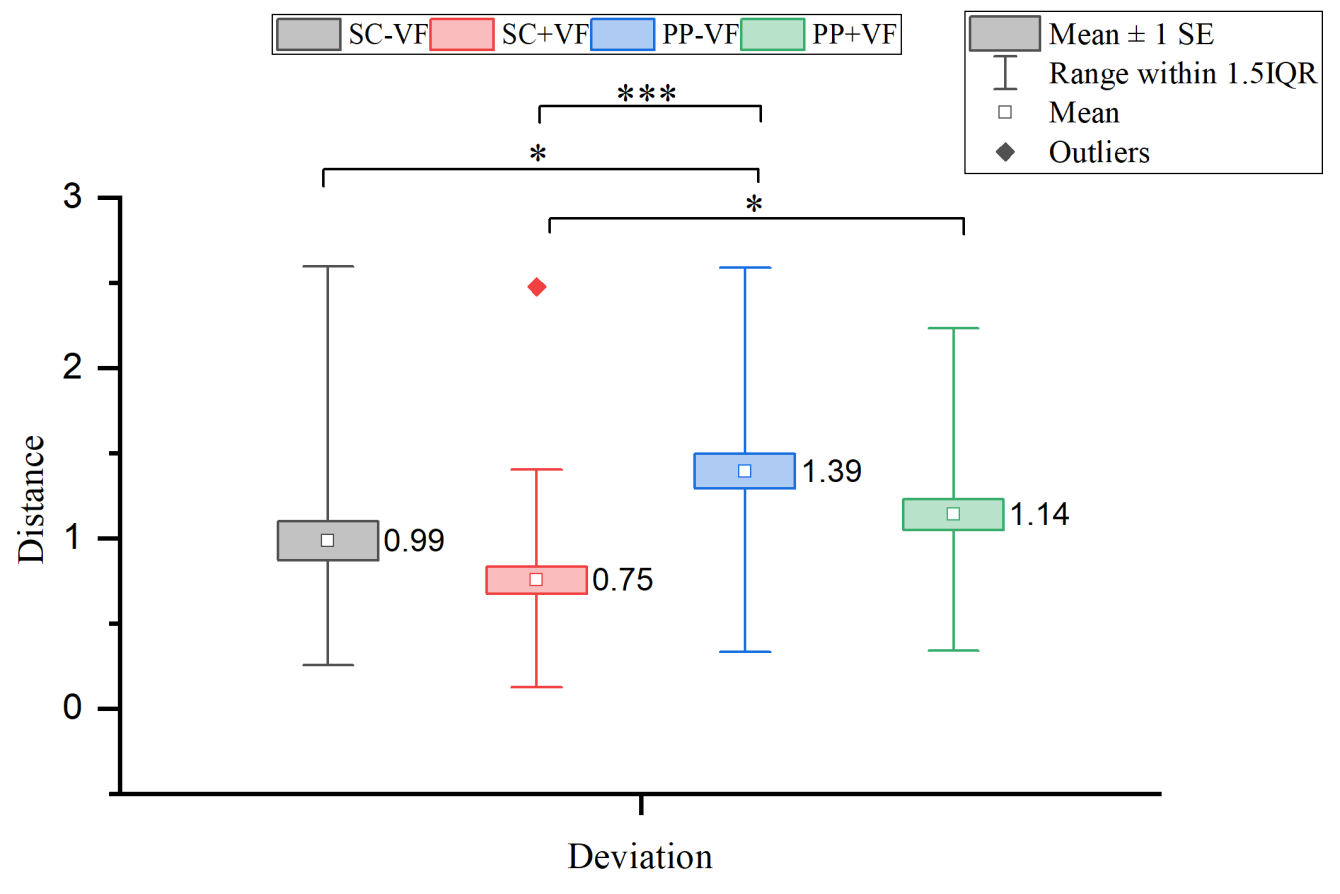
Results of comparisons of hit deviations. The square markers with different colors represent the outliers under different conditions, which are defined as data points located beyond 1.5 times the interquartile range from the quartiles, as indicated in the figure legend. In all figures, the symbols *,*** denote the levels of statistical significance after adjustments: * indicates an adjusted *p*-value < 0.05, and *** indicates an adjusted *p*-value < 0.001. *p*-values adjusted via the Bonferroni method for 6 comparisons ($p_{adj}=p_{original}\times 6$).

The Friedman test on the four experimental groups revealed significant between-group differences ($\chi^{2}\left(3\right)=24.055$, p<0.001). To identify the specific pairs that differed, Figure 6 displays the results of Post-hoc pairwise comparisons using Bonferroni correction. Specifically, the adjusted *p*-value ($p_{adj}$) was calculated as the original *p*-value multiplied by the number of pairwise comparisons (k = 6 for four conditions). The adjusted *p*-value was then compared directly to the significance level of α=0.05. Significant differences were observed between SC − VF and PP − VF (Z=−2.860, $p_{\mathrm{adj}}=0.025$), with the median hit deviation increasing by 79.9% when using physical props. Similarly, SC + VF and PP − VF showed a significant difference (Z=−4.767, $p_{\mathrm{adj}}<0.001$), while SC + VF and PP + VF also exhibited a significant discrepancy (Z=−3.051,$p_{\mathrm{adj}}=0.014$). Under visual feedback conditions, the median hit deviation increased by 59.5% due to the use of physical props.

#### 3.1.2. Task Completion Time Analysis

**Figure 7:**
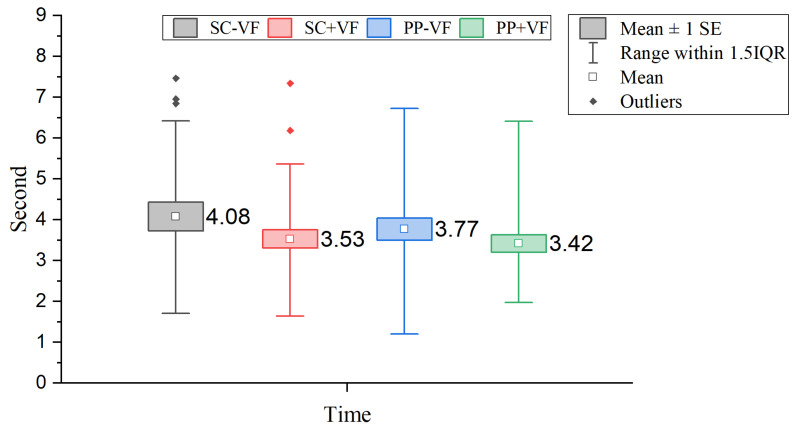
Results of comparisons of task completion time. The square markers with different colors represent the outliers under different conditions, which are defined as data points located beyond 1.5 times the interquartile range from the quartiles, as indicated in the figure legend.

We recorded and analyzed the task completion time for each participant across all four experimental conditions. As shown in Figure 7, the Friedman test revealed no statistically significant differences in task completion time among the four groups ($\chi^{2}\left(3\right)=3.436$, p>0.05).

#### 3.1.3. Hand Tremor Analysis

**Figure 8:**
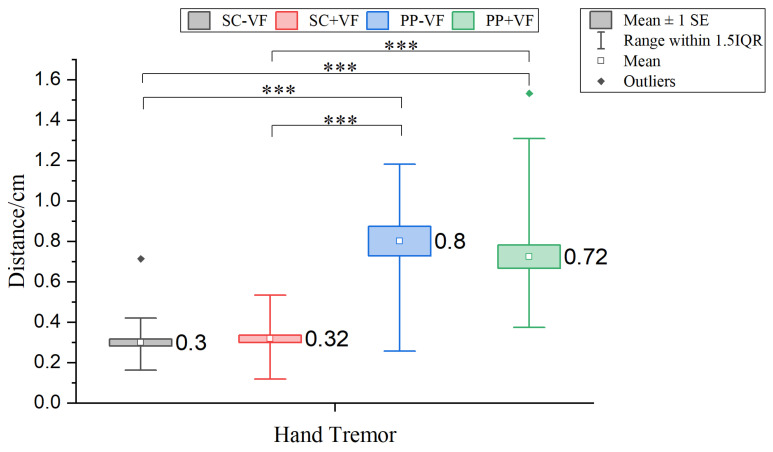
Results of comparisons of hand tremor. *** indicates an adjusted *p*-value < 0.001. *p*-values adjusted via the Bonferroni method for 6 comparisons ($p_{adj}=p_{original}\times 6$).

The difference in hand tremor across conditions was statistically significant ($\chi^{2}\left(3\right)=78.455$, p<0.001). As shown in Figure 8, the following groups showed significant differences in pairwise comparisons: SC − VF and PP − VF (Z=−7.056, $p_{\mathrm{adj}}<0.001$), SC + VF and PP + VF (Z=−5.339, $p_{\mathrm{adj}}<0.001$), SC − VF and PP + VF (Z=−6.484, $p=p_{\mathrm{adj}}<0.001$), and SC + VF and PP − VF (Z=−5.911, $p_{\mathrm{adj}}<0.001$). However, no significant differences were observed between the two SC-mode groups (Z=−1.144, $p_{\mathrm{adj}}>0.05$) or between the two PP-mode groups (Z=−0.572, $p_{\mathrm{adj}}>0.05$).

#### 3.1.4. Linear Mixed-Effects Analysis of Hand Tremor

These results from the non-parametric Friedman test provide an overall comparison of the conditions. However, to better understand the underlying factors and their interactions, we further conducted linear mixed-effects models, which allows us to control for variables and evaluate their independent contributions. A linear mixed-effects model was fitted to analyze the effects of visual feedback (VF), physical prop (PP), and time on hand tremor. The model included VF and PP as categorical fixed factors, Time as a continuous fixed covariate, and all their interactions. (No subjective experience variables from the questionnaires were included in this models.) Crucially, a random intercept for participants was included to account for individual differences in baseline hand tremor levels. The model was fitted using Restricted Maximum Likelihood (REML).

Type III tests of fixed effects revealed a significant main effect of PP (F(1, 113.79) = 70.59, *p* < 0.001) and Time (F(1, 119.93) = 8.50, *p* = 0.004). The main effect of VF remained non-significant (*p* = 0.477). A significant interaction was again found between PP and Time (F(1, 115.52) = 9.81, *p* = 0.002). None of the other interactions were significant (all *p* > 0.5).

The estimates labeled as [PP = 0] and [VF = 0] in the Table 2 represent the effects relative to their respective reference groups. The model estimates confirm that using a physical prop [PP = 1] is associated with significantly greater hand tremor than using the standard controller. The estimate for the standard controller group [PP = 0] (β = −1.216, *p* < 0.001), indicating a lower marginal mean for hand tremor. The significant interaction between [PP = 0] and time (β = 0.142, *p* = 0.014) suggests that the slope of hand tremor over time was significantly steeper for the group without physical props.

The variance component for the random intercept was statistically significant (*p* = 0.010), confirming that there were significant individual differences between participants in their baseline levels of hand tremor after accounting for fixed factors.

The results robustly indicate that the use of a physical prop is associated with significantly higher levels of hand tremor compared to the standard controller. This relationship is moderated by time, suggesting the effect of the interface type on hand tremor changes over the course of the task. Visual feedback show no significant effect.

#### 3.1.5. Linear Mixed-Effects Analysis of Hit Deviation

A linear mixed-effects model was fitted to analyze the factors that influence Hit Deviation. The model included Visual Feedback (VF) and Physical Prop (PP) as categorical fixed factors, time and hand tremor as continuous fixed covariates, and a critical interaction term VF × Hand Tremor. A random intercept for participants was incorporated to account for individual differences in baseline accuracy levels. The model was estimated using Restricted Maximum Likelihood (REML).

Type III tests of fixed effects revealed two significant effects: a significant main effect of Hand Tremor (F(1, 124.79) = 6.14, *p* = 0.015) and a significant interaction between VF and Hand Tremor (F(1, 101.13) = 7.01, *p* = 0.009). The main effects of VF (*p* = 0.174), PP (*p* = 0.495) and Time (*p* = 0.141) were not statistically significant, indicating no independent influence of these variables on Hit Deviation when controlling for other factors.

Parameter estimates in Table 3 further clarified the nature of the significant interaction. The coefficient for hand tremor (β = 0.552, *p* = 0.376) represents the simple slope for the reference group (VF = 1, visual feedback present), indicating non-significant association between hand tremor and hit deviation in this condition. Crucially, the interaction term [VF = 0] × Hand Tremor was highly significant (β = 1.517, *p* = 0.009), revealing that the effect of hand tremor on Hit Deviation was substantially stronger when visual feedback was absent. Specifically, for the VF-absent group (VF = 0), the total slope of hand tremor was calculated as 2.069 (=0.552 + 1.517), meaning that each one-unit increase in hand tremor was associated with an average increase of approximately 2.07 units in hit deviation—a relationship that was both strong and statistically significant.

The significant VF × Hand Tremor interaction indicates that visual feedback moderates the impact of physiological hand tremor on performance accuracy. When visual feedback is available, participants appear to compensate for hand movement, resulting in a minimal and non-significant effect of hand tremor on Hit Deviation. In contrast, in the absence of visual feedback, hand tremor exerts a pronounced detrimental effect on aiming precision, leading to a steep increase in error as instability increases.

The variance component for the random intercept was statistically significant (*p* = 0.013), confirming the presence of substantial individual differences in baseline hit deviation, even after accounting for the fixed effects.

The analysis revealed a significant moderating effect of interaction between visual feedback and Hand Tremor on the performance. While hand tremor significantly impairs performance, this negative effect is effectively buffered when visual feedback is available. The presence of visual feedback thus appears to enable compensatory motor control mechanisms that mitigate the impact of physical instability. Neither the type of physical prop nor the passage of time showed a significant direct effect on accuracy in this task context.

### 3.2. Questionnaire Result

To assess the reliability and validity of the measuring scales used in the study, reliability and validity analyses were conducted on the data collected from the four experimental groups. The analysis indicators included internal consistency reliability (Cronbach’s Alpha), the Kaiser-Meyer-Olkin (KMO) measure of sampling adequacy, and Bartlett’s Test of Sphericity. As shown in Table A1, the majority of the scales demonstrated good internal consistency (α > 0.7) and structural validity (KMO > 0.7, Bartlett’s Test of Sphericity significant at *p* < 0.05) across the different experimental conditions. This indicates that the measuring tools employed in this study possess high reliability and validity.

#### 3.2.1. Presence

**Figure 9:**
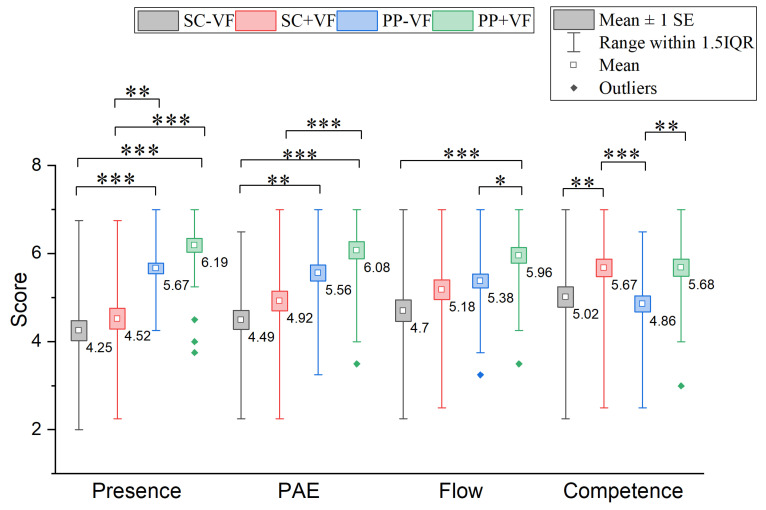
Results of comparisons of user experience. In the figure, * indicates an adjusted *p*-value < 0.05, ** indicates an adjusted *p*-value < 0.01, and *** indicates an adjusted *p*-value < 0.001. *p*-values adjusted via the Bonferroni method for 6 comparisons ($p_{adj}=p_{original}\times 6$).

The Friedman test indicated a statistically significant difference in presence among the four conditions ($\chi^{2}\left(3\right)=50.447$, p<0.001). As illustrated in Figure 9, post-hoc pairwise comparisons using Bonferroni correction showed significant differences between the SC − VF and PP − VF groups (Z=−4.577, $p_{\mathrm{adj}}<0.001$) and between the SC + VF and PP + VF groups (Z=−4.767, $p_{\mathrm{adj}}<0.001$), indicating that physical props significantly enhanced users’ sense of presence. Additionally, significant differences were found between SC − VF and PP + VF (Z=−6.007,$p_{\mathrm{adj}}<0.001$) and between SC + VF and PP − VF (Z=−3.337, $p_{\mathrm{adj}}=0.005$). However, no significant differences were detected between SC − VF and SC + VF (Z=−1.240, $p_{\mathrm{adj}}>0.05$) or between PP − VF and PP + VF (Z=−1.430, $p_{\mathrm{adj}}>0.05$), suggesting that physics-associated visual feedback did not significantly improve presence.

#### 3.2.2. PAE

A significant difference was found in PAE among the conditions($\chi^{2}\left(3\right)=41.621$, p<0.001). Follow-up pairwise comparisons, adjusted with Bonferroni correction, indicated significant differences between SC − VF and PP − VF (Z=−3.862, $p_{\mathrm{adj}}=0.001$) and between SC + VF and PP + VF (Z=−5.911, $p_{\mathrm{adj}}<0.001$), demonstrating that physical props effectively increased PAE. Differences between SC − VF and PP + VF (Z=−4.052, $p_{\mathrm{adj}}<0.001$) also reached statistical significance. No significant differences were found between SC − VF and SC + VF (Z=−1.859, $p_{\mathrm{adj}}>0.05$), SC + VF and PP − VF (Z=−2.002, $p_{\mathrm{adj}}>0.05$) or between PP − VF and PP + VF (Z=−2.050, $p_{\mathrm{adj}}>0.05$).

#### 3.2.3. Flow Experience

The Friedman test indicated a statistically significant difference in the flow experience among the four conditions ($\chi^{2}\left(3\right)=24.281$, p<0.001). Post-hoc analysis revealed that significant differences were only detected between SC − VF and PP + VF (Z=−4.577, $p_{\mathrm{adj}}<0.001$) and between PP − VF and PP + VF (Z=−2.717, $p_{\mathrm{adj}}=0.039$), highlighting the synergistic effect of physics-associated visual feedback on flow experience when combined with physical props. No significant differences were observed between SC − VF and SC + VF (Z=−2.145, $p_{\mathrm{adj}}>0.05$), SC − VF and PP − VF (Z=−1.859, $p_{\mathrm{adj}}>0.05$), SC + VF and PP − VF (Z=−0.286, $p_{\mathrm{adj}}>0.05$), or SC + VF and PP + VF (Z=−2.431, $p_{\mathrm{adj}}>0.05$).

#### 3.2.4. Competence

The Friedman test indicated a statistically significant difference in competence among the four conditions ($\chi^{2}\left(3\right)=26.526$, p<0.001). In the post-hoc analysis, significant differences were identified between SC − VF and SC + VF (Z=−3.575, $p_{\mathrm{adj}}=0.002$), SC + VF and PP − VF (Z=−4.243, $p_{\mathrm{adj}}<0.001$), and PP − VF and PP + VF (Z=−3.289, $p_{\mathrm{adj}}=0.006$), indicating that physics-associated visual feedback significantly enhanced perceived competence. Physical props alone showed no significant impact on percei- ved competence.

#### 3.2.5. SUS and Task Load

**Figure 10:**
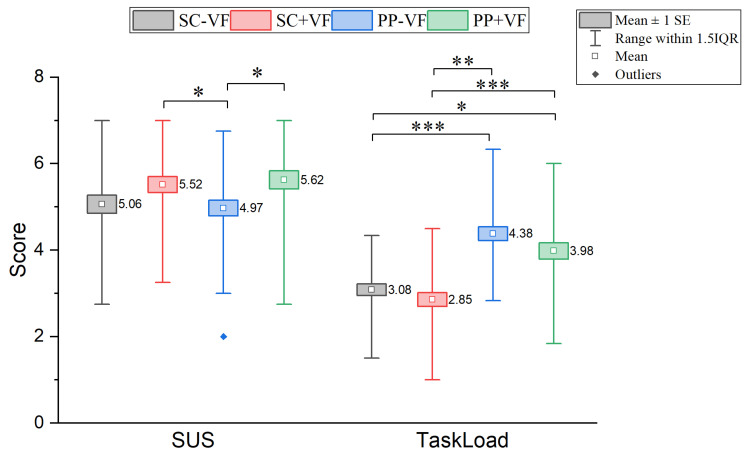
Results of comparisons of user task load and system useability. In the figure, * indicates an adjusted *p*-value < 0.05, ** indicates an adjusted *p*-value < 0.01, and *** indicates an adjusted *p*-value < 0.001. *p*-values adjusted via the Bonferroni method for 6 comparisons ($p_{adj}=p_{original}\times 6$).

In terms of system usability, the bow-and-arrow interaction system developed in this study demonstrated a good level of performance, as shown in Figure 10. According to the established SUS benchmark [44], the mean scores (converted to a 100-point scale) for all four experimental conditions ranged from 70.71 to 79.86). The Friedman test indicated a statistically significant difference in the system usability among the four conditions ($\chi^{2}\left(3\right)=13.087$, p=0.004). Significant differences in system usability were detected between SC + VF and PP − VF (Z=−2.670, $p_{\mathrm{adj}}=0.046$) and between PP − VF and PP + VF (Z=−2.765, $p_{\mathrm{adj}}=0.034$), suggesting that physics-associated visual feedback improves usability. This demonstrates that the introduced visual feedback, particularly when integrated with physical mechanics, is a key factor in effectively enhancing the system’s usability. Ultimately, the PP + VF condition achieved a score of 79.86, approaching the excellent level, which fully demonstrates to the success of the bow-and-arrow system’s design in meeting the requirements for good usability.

For task load, the Friedman test was significant ($\chi^{2}\left(3\right)=45.946$, p<0.001). Significant differences in task load were observed between SC − VF and PP − VF (Z=−4.910, $p_{\mathrm{adj}}<0.001$) and between SC + VF and PP + VF (Z=−3.957, $p_{\mathrm{adj}}<0.001$), confirming that physical props significantly increased task load. Differences between SC − VF and PP + VF (Z=−2.956, $p_{\mathrm{adj}}<0.019$) and SC + VF and PP − VF (Z=−5.911, $p_{\mathrm{adj}}<0.001$) were also significant. No differences were found between SC − VF and SC + VF (Z=−1.001, $p_{\mathrm{adj}}>0.05$) or PP − VF and PP + VF (Z=−1.955, $p_{\mathrm{adj}}>0.05$). This pattern confirms that physics-associated visual feedback did not contribute to higher task load. The TLX scores across experimental conditions were converted to a 0–100 scale for comparison with established reference values. The mean scores were as follows: SC − VF (44.00), SC + VF (40.71), PP − VF (62.57), and PP + VF (56.86). Relative to the meta-analytic reference values [45], where the overall mean TLX is 42 (SD = 13), the SC conditions align closely with the average workload range. In contrast, the PP conditions (56.86–62.57) exceed the 75th percentile (60.00) reported by Grier [46], indicating a high workload level. These scores are comparable to those observed in demanding tasks such as Physical Activities (median = 62.00) and Video Game tasks (median = 56.50) in Grier’s taxonomy. Overall, the task represents a moderate-to-high workload scenario, particularly when physical props are incorporated.

## 4. Discussion

### 4.1. Main Findings

The present study employed linear mixed-effects modeling to investigate the complex interplay between visual feedback (VF), physical prop (PP), task completion time, hand tremor, and hit deviation. By analyzing two distinct but interrelated models—one predicting hand tremor and the other predicting Hit Deviation—we uncover a nuanced, moderated pathway through which motor control dynamics influence performance outcomes in a simulated archery task.

First, analysis of hand tremor revealed that the use of a physical prop exerts a powerful influence on hand stability. Participants exhibited significantly lower levels of hand tremor when using the standard controller compared to the physical prop, indicating that the latter may introduce biomechanical constraints or unfamiliar motor demands that amplify tremor. Furthermore, a significant Physical Prop × Time interaction demonstrated that the trajectory of hand stability over time differs markedly between interface types. Specifically, hand tremor decreased over time in physical prop condition. This pattern suggests that the physical prop, by providing richer kinesthetic feedback—including greater perceived weight, inertia, and continuous mechanical constraints—may support more effective real-time sensorimotor compensation for hand tremor, enabling users to dynamically suppress tremor during task execution. In contrast, no such improvement was observed with the standard controller, where hand tremor remained relatively stable or even slightly increased. Notably, visual feedback did not significantly affect hand tremor, either as a main effect or in interaction with other variables, indicating that visual feedback does not directly modulate motoric tremor in this context.

Second, and more critically, the analysis of Hit Deviation revealed that hand tremor impacts hit deviation—but this relationship is strongly contingent on the availability of visual feedback. When visual feedback was present (VF = 1), the slope of the hand tremor–hit deviation relationship was weak and non-significant (β=0.552, p=0.376), suggesting that real-time visual information enables participants to compensate for motor instability—likely through real-time sensorimotor compensation. However, when visual feedback was absent (VF = 0), the total effect of hand tremor on hit deviation became substantially stronger (β=2.069), and highly significant (p=0.009). This indicates that without external visual guidance, individuals are unable to mitigate the negative consequences of physiological tremor, leading to a direct transmission of motor noise into performance error.

### 4.2. Reconciling Findings from Different Analytical Approaches

It is important to address the apparent discrepancies between Post-hoc pairwise comparisons and the linear mixed-effects models by recognizing their distinct analytical perspectives. The Post-hoc pairwise comparisons evaluates the total effect of experimental conditions, while the linear mixed-effects model addresses a more refined question: it isolates the independent, direct contribution of each factor after accounting for the influence of other variables. This distinction resolves the two key observations.

First, the significant difference in hit deviation between the SC + VF and PP + VF conditions identified by Post-hoc pairwise comparisons using Bonferroni correction (Z=−3.051, $p_{\mathrm{adj}}=0.014$) reflects the genuine total detriment associated with the physical prop. The linear mixed-effects model clarifies that this total effect is predominantly mediated by an indirect pathway: the prop significantly increases hand tremor, which in turn degrades aiming precision. When this physiological mediator is statistically controlled, the direct path from the prop to hit deviation becomes non-significant.

Second, the absence of a simple main effect of visual feedback (VF) on Hit Deviation in the Post-hoc pairwise comparisons aligns with its role as a moderator in the linear mixed-effects model. The model identified a significant VF × Hand Tremor interaction, indicating that VF’s benefit is contingent upon the level of physiological instability; it functions specifically to attenuate the deleterious impact of hand tremor. Thus, although visual feedback attenuates the impact of hand tremor on performance, it does not fully eliminate the indirect negative consequences of using the physical prop. Even under VF, participants using the physical prop experienced substantially higher levels of hand tremor, and although the sensorimotor system could partially compensate, the cumulative effect of increased motor noise translated into measurably higher hit deviations. The moderating role of VF therefore reduces—but does not nullify—the indirect pathway from PP to performance degradation via increased physiological instability.

### 4.3. Design Inspiration

**Figure 11:**
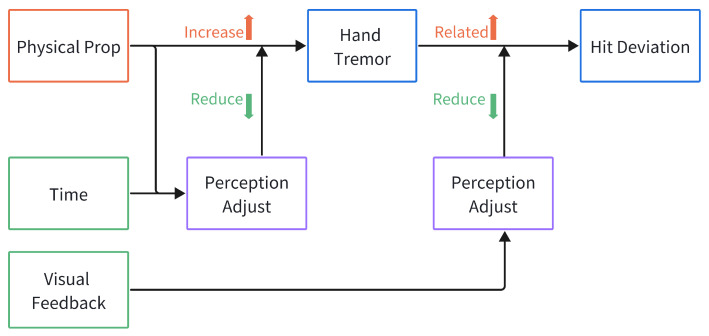
Dependent causal chain while using visual feedback and physical prop.

Together, these findings trace a clear and conditionally dependent causal chain when visual feedback (VF) and physical prop (PP) are used in combination, as shown in Figure 11: The introduction of the specific physical bow prop considerably reduces the performance of the user’s task. This is due to the demands of the archery task on precise technique and force control, where the weight of the real bow and string vibrations increases operational difficulty, leading to exacerbated hand tremor that impair fine motor execution. Notably, this effect is modulated by time—users exhibit a gradual reduction in hand tremor over time when using the physical prop, suggesting partial adaptation to its biomechanical demands. Visual feedback determines its propagation into task performance. These findings have practical implications for the design of human–computer interaction systems—particularly in virtual reality and precision skilled tasks—where minimizing reliance on continuous visual feedback may require additional strategies to stabilize motor output or provide alternative sensory cues.

Although physical props reduced task performance, they markedly enhanced users’ sense of presence and physical activity enjoyment. Experimental results indicate that physical feedback from physical props amplified users’ positive emotional engagement. However, physical props also resulted in higher task load. Therefore, in such high-skill tasks, the application of Physical props requires considering user capability.

The physics-associated visual feedback exhibited an important role in the experiment: The enhancement of flow experience through visual feedback was limited under standard controller conditions, showing discernible effects only when combined with physical props. In other words, when using standard controller, physics-associated visual feedback cannot compensate for sensory dissonance or improve users’ flow experience. This indicates that the utility of physics-associated visual feedback is highly dependent on the continuous action-feedback cognitive loop under physical props conditions.

In addition, regarding users’ self-reported intention for future use, we observed a descriptive trend: participants reported a higher willingness to continue using the system after experiencing the condition involving ‘physical props combined with visual feedback.’ Although this was measured using only a single item, the trend is consistent with users’ positive feedback on physical activity enjoyment, flow experience. This further suggests, from an applied perspective, that despite the increased operational demands associated with physical props, the heightened immersion and the synergistic positive experience generated with visual feedback may enhance the system’s long-term appeal and user retention.

### 4.4. Limitations and Future Work

Sample Size and Population: Although our sample of 33 university students aligns with common practices in the field and significant effects were detected, we acknowledge that a larger sample could reveal more subtle effects. The predominantly young, technologically literate participant pool may not represent broader populations, particularly individuals with different skill levels. Since most of the participants were beginners, our findings have certain limitations for users with different archery skill levels. Future research should systematically examine how skill level modulates the effects of props and visual feedback.

Gesture Recognition System Limitations: The computer vision-based hand tracking system, while cost-effective, introduced several limitations in tracking accuracy and latency. Two key technical challenges were identified: (1) real-world 2D key point detection introduced significant noise that propagated through the pipeline; (2) the test system assumed optimal hand visibility and recognition conditions, which may fail in complex real-world environments with occlusions or varying lighting. Future research could employ more precise tracking systems, such as advanced depth sensors, combined with multi-sensor information fusion technology to more accurately capture subtle movements and behaviors while improving robustness to environmental variations.

The Specific Characteristics of the Physical Props: The study used a single physical bow. Different prop characteristics (vibration, weight distribution, size, material properties) might produce varying effects on both performance and experience. Future work should systematically examine how specific prop parameters influence the observed trade-offs.

Interpretation of Visual Feedback: Our study did not directly assess how users cognitively interpreted the visual feedback. This limits our understanding of the users’ mental models. To address this, future work could focus on investigating how users understand and interpret trajectory lines in visual feedback.

## 5. Conclusions

This study developed a sensor-driven framework for investigating high-skill tasks in VR. By integrating a vision sensor with standard VR devices, we moved beyond subjective metrics to objectively capture and analyze performance data, such as hand tremor and operation time. This approach allowed us to elucidate the underlying mechanisms through which physical prop interfaces and visual feedback interact to shape user performance in VR archery. It demonstrates how strategically combining available sensing technologies can yield new insights into human-computer interaction, particularly in understanding the complex relationship between interface design, motor performance, and user experience.

The User experience study shows that, the proposed VR archery interaction framework, which is driven by sensors and physical props, enhanced user’s presence and enjoyment but also affected the task performance. More importantly, by objectively quantifying performance with the proposed sensor-driven VR framework, we reveal the dual-pathway mechanism. Although physics-associated visual feedback doesn’t reduce hand tremor directly, it enables sensorimotor compensation that effectively buffers against motor instability. This compensatory mechanism reveals that visual feedback serves as a crucial moderator in the pathway from physical interface to performance outcomes.

Furthermore, visual feedback demonstrates synergistic effects with physical props, particularly in enhancing flow experience and perceived competence. This multimodal integration improves the user experience by creating a more engaging and effective interaction paradigm through the combination of rich kinesthetic feedback with congruent visual cues.

In summary, this study contributes to VR interaction research by introducing a new sensor-driven methodological framework. The framework developed in this study enabled a mechanistic understanding of VR interaction. We objectively demonstrated that physical props increase hand tremor, thereby impairing performance, while physics-associated visual feedback mitigates this effect by enabling sensorimotor compensation. The proposed VR archery framework driven by sensors and physical props and the accompanying user study provide a foundation for future work aimed at designing immersive, effective, and accessible VR systems that leverage sensors and physical interfaces. 

## Figures and Tables

**Table 1:** Summary of Measurement Variables.

Category	Variable	Measurement Scale
Objective	Hit Deviation	Normalized distance
	Hand Tremor	Centimeters
	Task Completion Time	Seconds
Subjective	Presence	Likert-7
	Physical Activity Enjoyment (PAE)	Likert-7
	Flow Experience	Likert-7
	Competence	Likert-7
	Task Load	Likert-7
	System Usability	Likert-7
	Future Use Intention	Likert-7

**Table 2:** Fixed Effects Estimates-Parameter Coefficients for Hand Tremor Model.

Parameter	Estimate	Significance
Intercept	2.132	p<0.001 *** ^1^
[VF = 0] ^2^	0.055	p=0.775
[PP = 0]	−1.216	p<0.001 ***
Time	−0.112	p=0.008 **
[PP = 0] × Time	0.142	p=0.014 *

^1^ In the table, * indicates an adjusted *p*-value < 0.05, ** indicates an adjusted *p*-value < 0.01, and *** indicates an adjusted *p*-value < 0.001. *p*-values adjusted via the Bonferroni method for 6 comparisons ($p_{adj}=p_{original}\times 6$). ^2^ It should be noted that categorical variables were dummy-coded with the level “1” set as the reference group: PP = 1 denotes the use of the physical prop, and PP = 0 denotes the use of the standard controller; VF = 1 denotes the presence of visual feedback, and VF = 0 denotes the absence of visual feedback.

**Table 3:** Fixed Effects Estimates-Parameter Coefficients for Hit Deviation Model.

Parameter	Estimate	Significance
Intercept	0.457	p=0.705
[VF = 0]	−0.115	p=0.174
[PP = 0]	−0.337	p=0.495
Time	0.163	p=0.141
Hand Tremor	0.552	p=0.376
[VF = 0] × Hand Tremor	1.517	*p* = 0.009 **^1^

^1^ In the table, ** indicates an adjusted *p*-value < 0.01. *p*-values adjusted via the Bonferroni method for 6 comparisons ($p_{adj}=p_{original}\times 6$).

## Data Availability

The data that support the findings of this study are available from the corresponding author upon reasonable request.

## Appendix A

**Table A1:** Reliability and Validity Test Results.

Measurement	Model	Cronbach’s Alpha	KMO	Bartlett’s Test of Sphericity		
				Chi-Square	df	Sig.
Presence	SC − VF	0.909	0.822	89.463	6	0.000
	SC + VF	0.911	0.821	86.018	6	0.000
	PP − VF	0.700	0.734	23.150	6	0.001
	PP + VF	0.883	0.758	70.769	6	0.000
PAE	SC − VF	0.828	0.768	59.059	6	0.000
	SC + VF	0.876	0.816	68.78	6	0.000
	PP − VF	0.842	0.797	51.496	6	0.000
	PP + VF	0.855	0.795	87.658	6	0.000
Flow Experience	SC − VF	0.896	0.747	82.538	6	0.000
	SC + VF	0.884	0.713	88.956	6	0.000
	PP − VF	0.697	0.703	27.851	6	0.000
	PP + VF	0.781	0.721	59.258	6	0.000
Competence	SC − VF	0.904	0.831	80.198	6	0.000
	SC + VF	0.892	0.804	83.697	6	0.000
	PP − VF	0.873	0.792	65.136	6	0.000
	PP + VF	0.907	0.830	88.088	6	0.000
